# Recurrence of sexually transmitted infections is commonly found in a subpopulation of Austrian users of HIV pre-exposure prophylaxis

**DOI:** 10.1007/s00508-025-02499-6

**Published:** 2025-03-02

**Authors:** Nikolaus Urban, Thomas Neidhart, Katharina Grabmeier-Pfistershammer, Veronique Touzeau-Roemer, Kaspar Laurenz Schmidt, Robert Strassl, Wolfgang Weninger, Birgit Willinger, Wolfgang Michael Bauer, David Chromy

**Affiliations:** 1 Department of Dermatology, Medical University of Vienna, Vienna, Austria; 2 Institute of Clinical Virology, Department of Laboratory Medicine, Medical University of Vienna, Vienna, Austria; 3 Division of Clinical Microbiology, Department of Laboratory Medicine, Medical University of Vienna, Vienna, Austria

**Keywords:** Men who have sex with men, Sexually transmitted infections, Human immunodeficiency virus, Pre-exposure prophylaxis, Chemsex

## Abstract

**Background:**

In recent years there has been an increase in the diagnoses of sexually transmitted infections (STI) among men who have sex with men (MSM) using human immunodeficiency virus (HIV) pre-exposure prophylaxis (PrEP); however, data on PrEP users in Austria are limited.

**Patients, material and methods:**

In June 2020, we initiated a prospective observational cohort study at Vienna General Hospital including PrEP users from Vienna. Participants underwent STI testing quarterly and provided behavioral information using a questionnaire.

**Results:**

Between June 2020 and December 2023 a total of 360 individuals (99% MSM) were enrolled comprising 379 person-years of follow-up. We identified 276 STIs in 154 individuals, of which 23% (36/154) were symptomatic. The incidence rates per 100 person-years were 29.9 (95% confidence interval, CI 24.3–35.3 per 100 person-years) for gonorrhea, 22.7 (95% CI 17.9–27.5 per 100 person-years) for chlamydia and 9.8 (95% CI 6.6–12.9 per 100 person-years) for syphilis. Extragenital infections accounted for 95% (97/102) of gonorrhea and 81% (65/80) of chlamydia cases. A case of HIV infection was recorded in a 20-year-old male with inconsistent PrEP use. Participants with one or more reinfection (18%; 65/360) accounted for 68% (187/276) of all STIs. Sexualized drug use (Chemsex) was reported by 44% (157/360) of participants and was significantly associated with higher rates of gonorrhea (38% vs. 21%, *p* < 0.001) and syphilis (17% vs. 5%, *p* < 0.001) but not chlamydia (26% vs. 19%, *p* = 0.118).

**Conclusion:**

Throughout the study, 43% of participants experienced a bacterial STI, which was mostly asymptomatic and at extragenital sites. Chemsex was commonly reported and identified as a predictor for STI reinfection, underlining the importance of harm reduction strategies in Austrian STI prevention.

**Supplementary Information:**

The online version of this article (10.1007/s00508-025-02499-6) contains supplementary material, which is available to authorized users.

## Introduction

Human immunodeficiency virus (HIV) pre-exposure prophylaxis (PrEP) has emerged as a highly effective and safe HIV prevention strategy [1, 2]; however, its implementation has been accompanied by frequent screening for sexually transmitted infections (STIs), such as gonorrhea, chlamydia and syphilis [3], causing a rise in STI incidence [4]. Men who have sex with men (MSM) are the primary target group for PrEP services in countries like Austria and Germany [5] as, compared to the general population, they are more likely to engage in condomless anal intercourse (CAI) with casual partners [6] as well as in sexualized drug use (chemsex). Chemsex has experienced a growing prevalence in many western countries [7, 8], involves the use of recreational drugs like mephedrone or gamma-hydroxybutyric acid to enhance sexual experiences and has been linked to transmission-prone sex practices like CAI. Furthermore, chemsex is associated with a higher STI prevalence [9, 10]. In Austria, PrEP became widely accessible in 2018 [11]; however, despite a substantial number of individuals meeting PrEP eligibility criteria, its rollout has been hampered by limited PrEP service availability and delayed coverage by the healthcare providers [12]. Comprehensive data regarding characteristics, sexual risk behavior and STI prevalence and incidence among PrEP users in Austria have been scarce. This study aimed to address these gaps by investigating Austrian PrEP users longitudinally. These findings can inform PrEP services and healthcare authorities on current challenges in Austrian PrEP users and serve as a baseline for the upcoming era of doxycycline postexposure prophylaxis (Doxy PEP).

## Patients, material and methods

### Study design and participants

This prospective observational cohort study was initiated in June 2020 at the HIV and STI outpatient clinic of the Department of Dermatology at the General Hospital of Vienna. All persons living without HIV who presented at the clinic were assessed for PrEP eligibility. Participants were interviewed about their medical history, with additional information collected from previous medical records when available. The inclusion criteria included MSM and transgender individuals living without HIV, aged 18 years or older, who met one or more of the following conditions: (i) inconsistent condom use with casual partners and/or partners living with HIV not on treatment, (ii) recent acquisition of an STI, (iii) recent use of HIV postexposure prophylaxis (PEP) and/or (iv) frequent engagement in chemsex within the past 12 months. All individuals were counselled and offered PrEP initiation or continuation. Both daily and on-demand PrEP were offered. Participants were subsequently monitored every 3 months. This work presents data from participants enrolled and monitored between June 2020 and December 2023. The study complied with the ethical standards of the Declaration of Helsinki and received approval from the local ethics committee of the Medical University of Vienna (MUW-EK 1051-2020). All participants provided both verbal and written informed consent.

### Parameters

All symptomatic and asymptomatic patients underwent physical examination and screening for STIs, including sampling of pharyngeal and anal swabs and native urine collection for nucleic acid amplification technology (NAAT) testing with commercially available test kits for *Neisseria gonorrhoeae *(NG) and *Chlamydia trachomatis *(CT) (BD MAX^TM^BD Molecular Diagnostics. Sparks. United States). Additionally, serological testing for HIV, hepatitis B virus, hepatitis C virus (HCV), and syphilis, along with PCR testing for HIV and HCV, were performed.

Participants completed a self-administered questionnaire, addressing sexual behavior over the past 12 (baseline questionnaire) or 3 months (3-month visit questionnaire), including preferences for on-demand versus daily PrEP, number of sex partners, sex practices, anal bleeding after intercourse and sexualized drug use.

### Outcome and definitions

The primary outcome of this study was the incidence of STIs in Austrian PrEP users. Previous PEP and PrEP usage and past STIs were documented based on the patient’s medical history. Symptomatic disease was defined by the presence of at least one of the following clinical signs: sore throat, oral or genital ulcerations, urethral pain or discharge, anal pain or discomfort, or swollen lymph nodes. Active syphilis infection was determined by a positive Treponema pallidum particle agglutination assay (TPPA) combined with a reactive Venereal Disease Research Laboratory (VDRL) test, or a positive TPPA with a non-reactive VDRL test in the absence of prior treatment for syphilis. The presence of any STI was defined by any positive NAAT result for NG, CT, or a positive test indicating active syphilis infection. While reinfection was characterized by the detection of an additional infection with any STI at a subsequent visit, history of NG/CT/syphilis was defined as having been diagnosed with the respective STI at least once before enrolment, either as reported by the individual or documented in past medical records.

### Statistical analysis

Statistical analyses were conducted using GraphPad Prism 8 (GraphPad Software, La Jolla, CA, USA) and IBM SPSS Statistics 28 (IBM, Armonk, NY, USA). Histograms were used to visualize continuous variables and assess Gaussian normal distributions. Continuous variables were expressed as mean ± standard deviation or, for non-parametric distributions, as median and interquartile range (IQR, 25–75th percentiles). Nominal variables were presented as numbers and percentages. Group comparisons for continuous variables were performed using the Wilcoxon-Mann-Whitney U test or Student’s t‑test, depending on the distribution of data. Categorical variables were compared using Pearson’s χ^2^-test or Fisher’s exact test as appropriate. Binary logistic regression models were employed to analyze factors associated with dichotomous outcomes, with results reported as odds ratios (OR) and 95% confidence intervals (CI). Statistical significance was defined as *p* < 0.05 for all analyses.

## Results

### Demographics and individual history

A total of 360 participants were enrolled in the study, with 99% identifying as MSM and 1 person as a transgender woman. The median age of participants was 31.2 years (IQR 11.6 years). Of the individuals 58% preferred daily PrEP, while 42% opted for on-demand PrEP (Table 1). Previous use of PEP was reported by 22% of participants and 36% had prior experience with PrEP. At baseline, 23%, 26%, and 16% of participants had a history of syphilis, gonorrhea, and chlamydia, respectively. Chemsex was reported by 44% (157/360) of participants, of whom 64% (101/157) preferred daily PrEP compared to 53% (109/203) of those who did not engage in chemsex (*p* = 0.045). Additionally, 41% (64/156) of chemsex users had a history of PrEP use, compared to 32% (64/198) of non-users (*p* = 0.091).

**Table 1:** Individual history and sexually transmitted infections

	All individuals	No reported chemsex use	Reported chemsex use	*p*-value
	*N* = 360	*N* = 203	*N* = 157	
MSM (%, *n*/all)	99% (359/360)	100% (203/203)	99% (156/157)	–
Transgender women (%, *n*/all)	1% (1/360)	0% (0/203)	1% (1/157)	
Preference for daily PrEP (%, *n*/all)	58% (210/360)	53% (109/203)	64% (101/157)	0.045
Preference for on-demand PrEP (%, *n*/all)	42% (150/360)	47% (94/203)	36% (56/157)	
Median age (years) (IQR)	31.2 (11.6)	30.9 (10.7)	31.6 (12.5)	0.664
Previous PEP use (%, *n*/all)	22% (80/359)	23% (47/202)	21% (33/157)	0.612
Previous PrEP use (%, *n*/all)	36% (128/354)	32% (64/198)	41% (64/156)	0.091
*History of STIs prior to inclusion (%, n/all)*				
Syphilis	23% (82/359)	18% (36/202)	29% (46/157)	0.010
Gonorrhea	26% (94/357)	19% (39/201)	35% (55/156)	<0.001
Chlamydia	16% (57/357)	13% (27/201)	19% (30/156)	0.138
*Individuals affected at least once by the respective sexually transmitted infection during follow-up (%, n/all)*				
Any infection§	43% (154/360)	33% (67/203)	55% (87/157)	<0.001
Symptomatic disease	23% (36/154)	22% (15/67)	24% (21/87)	0.799
Median count of any STI (range)	1 (4)	1 (4)	1 (4)	–
Any reinfection	42% (65/154)	34% (23/67)	48% (42/87)	0.082
Syphilis	11% (38/360)	5% (11/203)	17% (27/157)	<0.001
Symptomatic	16% (6/38)	9% (1/11)	19% (5/27)	0.650
Gonorrhea	28% (102/360)	21% (42/203)	38% (60/157)	<0.001
Pharyngeal infection	18% (66/360)	16% (33/203)	21% (33/157)	0.247
Symptomatic	6% (4/66)	6% (2/33)	6% (2/33)	–
Urethral infection	7% (25/360)	3% (7/203)	11% (18/157)	0.003
Symptomatic	56% (14/25)	43% (3/7)	61% (11/18)	0.656
Anal infection	19% (67/360)	10% (20/203)	30% (47/157)	<0.001
Symptomatic	25% (17/67)	30% (6/20)	23% (11/47)	0.558
Chlamydia	22% (80/360)	19% (39/203)	26% (41/157)	0.118
Pharyngeal infection	3% (11/360)	2% (5/203)	4% (6/157)	0.458
Symptomatic	9% (1/11)	20% (1/5)	0% (0/6)	0.455
Urethral infection	5% (19/360)	5% (11/203)	5% (8/157)	0.892
Symptomatic	26% (5/19)	18% (2/11)	38% (3/8)	0.603
Anal infection	17% (62/360)	15% (30/203)	20% (32/157)	0.163
Symptomatic	24% (15/62)	37% (11/30)	13% (4/32)	0.038

§comprises all gonorrhea, chlamydia and syphilis infections

*IQR* interquartile range; *MSM* men who have sex with men; *PEP* HIV postexposure prophylaxis; *PrEP* HIV pre-exposure prophylaxis; *STI* sexually transmitted infection, *HIV* human immunodeficiency virus

### Sexually transmitted infections and risk behavior

**Fig. 1:**
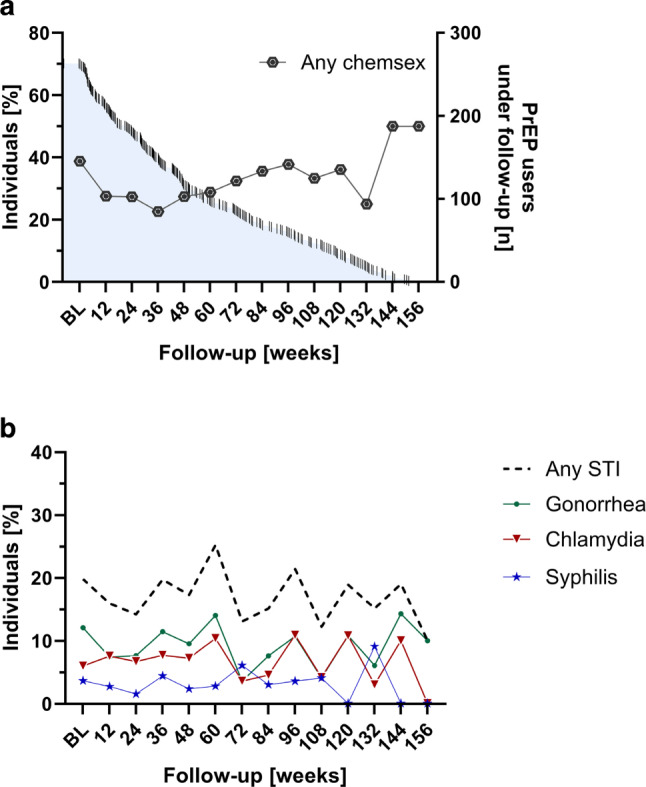
Chemsex and STI incidence during the observation period. **a** Chemsex engagement during the follow-up period is depicted. **b** Incidence of any STI, gonorrhea, chlamydia, and syphilis during the follow-up period is illustrated

Out of all participants enrolled 43% (154/360) tested positive for at least 1 STI, with 23% (36/154) of these cases presenting with symptoms. Gonococcal urethritis was frequently (56%, 14/25) associated with symptoms, whereas one quarter of individuals with anal gonorrhea (25%, 17/67) or chlamydia (24%, 15/62) experienced symptoms of proctitis. Chemsex users exhibited a significantly higher rate of STIs compared to non-users (55%, 87/157, vs. 33%, 67/203; *p* < 0.001; Table 1). Among those affected, the rates of specific infections were as follows: syphilis affected 11% (38/360), gonorrhea 28% (102/360) and chlamydia 22% (80/360) of participants. The rectal mucosa was the most common infection site for both gonorrhea and chlamydia (19%, 67/360, and 17%, 62/360, of all infections, respectively). Anal infections with gonorrhea were notably higher among chemsex users compared to non-chemsex users (30%, 47/157, vs. 10%, 20/203; *p* < 0.001). Similarly, statistically significant differences were observed in urethral infections (11%, 18/157 vs. 3%, 7/203; *p* = 0.003) but not in pharyngeal infections (21%, 33/157 vs. 16%, 33/203, *p* = 0.247); however, in the case of chlamydia there was no statistically significant difference between chemsex and non-chemsex users. Furthermore, 42% (65/154) of participants who tested positive for an STI during follow-up experienced at least 1 reinfection. Participants with 1 or more reinfections (18%, 65/360) accounted for 68% (187/276) of all detected STIs over the study period (Fig. 1).

### Incidence of sexually transmitted infections

Out of 360 participants enrolled at baseline, 263 attended at least 1 consecutive follow-up visit and were therefore included in the follow-up analysis during the study period from June 2020 to December 2023, contributing a total of 379 person-years of observation (Table 2).

**Table 2:** Incidence of STIs

**Follow-up**		
*June 2020–December 2023*		
Included individuals	*N* = 359	
Individuals with follow-up	*N* = 263	
Patient-years follow-up	379	
**Incidence of infections per 100 patient-years**		95% confidence interval
HIV	0.0	–
Hepatitis C virus infections	0.0	–
Gonorrhea	29.8	24.3–35.3
Chlamydia	22.7	17.9–27.5
Syphilis	9.8	6.6–12.9

**Fig. 2:**
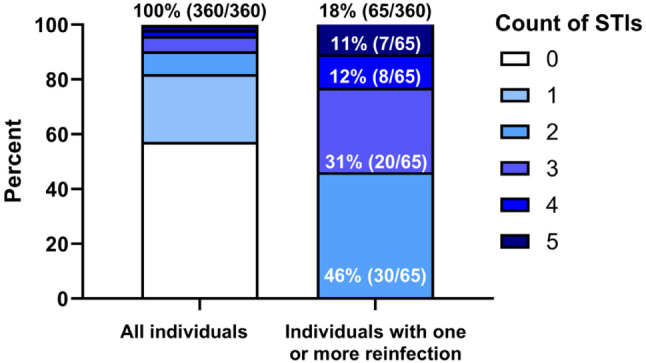
STI infections and reinfections. The left box displays the entire study population stratified by the count of sexually transmitted infections, whereas the right box provides a detailed view on those affected by one or more reinfections during follow-up. Notably, the cumulative number of STIs was 276, yet individuals with one or more reinfection (18%, 65/360) accounted for 68% (187/276) of them

The incidence rates of STIs per 100 person-years were as follows: gonorrhea 29.8 (95% CI: 24.3–35.3), chlamydia 22.7 (95% CI: 17.9–27.5) and syphilis 9.8 (95% CI: 6.6–12.9). Notably, one HIV infection was recorded during the study period in a 20-year-old male with inconsistent PrEP use. No HCV infection was detected. Figure 2b illustrates the prevalence of chemsex and the incidence of respective STIs during the observation period.

### Predictors for reinfection

A binary logistic regression model was used to predict the occurrence of reinfections with any STI (Table 3). In the univariate analysis, a preference for daily PrEP (OR 2.47, 95% CI: 1.34–4.56, *p* = 0.004), previous PrEP use (OR 2.28, 95% CI: 1.32–3.93, *p* = 0.003), and engagement in chemsex (OR 2.86, 95% CI: 1.63–5.00, *p* < 0.001) were found as positive predictors for STI reinfection; however, in multivariate analysis chemsex remained the only predictor for STI reinfection (adjusted OR 3.34, 95% CI: 1.67–6.65, *p* < 0.001).

**Table 3:** Predictors for any STI reinfection

	All individuals	All individuals without reinfections	All individuals with reinfections	Odds ratio	*p*-value	Adjusted odds ratio§	*p*-value
	*N* = 360	*N* = 295	*N* = 65	(95% CI)		(95% CI)	
*Daily PrEP preferred*	57% (202/352)	54% (156/290)	74% (46/62)	2.47 (1.34–4.56)	**0.004**	1.52 (0.72–3.21)	0.269
*Age*							
≤24 years	17% (62/360)	18% (54/295)	12% (8/65)	–	–	–	–
25–34 years	46% (164/360)	45% (133/295)	48% (31/65)	1.57 (0.68–3.64)	0.290	1.07 (0.37–3.06)	0.905
≥35 years	37% (134/360)	37% (108/295)	40% (26/65)	1.63 (0.69–3.83)	0.267	0.45 (0.15–1.41)	0.171
*Previous PEP use*	22% (80/359)	23% (69/294)	17% (11/65)	0.66 (0.33–1.34)	0.254	0.93 (0.38–2.29)	0.873
*Previous PrEP use*	36% (128/354)	33% (94/289)	52% (34/65)	2.28 (1.32–3.93)	**0.003**	1.12 (0.55–2.30)	0.750
*Any chemsex*	44% (157/360)	39% (115/295)	65% (42/65)	2.86 (1.63–5.00)	**<0.001**	3.34 (1.67–6.65)	**<0.001**

§ the binary logistic regression model included all variables investigated in this table and a correction for the time of follow-up

*CI* confidence interval, *PEP* HIV postexposure prophylaxis, *PrEP* HIV pre-exposure prophylaxis, *HIV* human immunodeficiency virus

## Discussion

In this prospective observational cohort study, 360 PrEP users were included with 263 being followed over 379 person-years. A high incidence of mostly asymptomatic STIs among PrEP users was found and chemsex users were disproportionately affected. Furthermore, study participants frequently engaged in transmission-prone sex practices, including chemsex and CAI. Ultimately, chemsex was identified as a predictor for STI reinfection among PrEP users.

In our cohort, STI incidence rates were relatively high, with gonorrhea at 29.8, chlamydia at 22.7, and syphilis at 9.8 per 100 person-years. These findings emphasize the substantial risk of bacterial STIs among PrEP users despite effective HIV prevention. Our findings are supported by other studies on PrEP users. For example, in a meta-analysis Werner et al. reported pooled incidence estimates of gonorrhea, chlamydia, and syphilis at 39.6, 41.8, and 9.1 per 100 person-years, respectively [13]. Comparable results were found in cohort studies from Australia [4] and the Netherlands [14], underlining the high burden of bacterial STIs in this population. Additionally, we identified a new HIV infection in our cohort of PrEP users, which occurred in an individual with inconsistent adherence to PrEP. This is one more unfortunate example that the effectiveness of oral PrEP is mainly limited by adherence and not the regimen’s efficacy. Accordingly, PrEP users should receive frequent counselling on the importance of consistent PrEP use.

In our cohort, 44% of participants reported chemsex use. This is in line with findings from Hoornenborg et al., who reported a chemsex prevalence of 41% in the AMPrEP study in the Netherlands [15], and Anato et al., who found a prevalence of 24% among PrEP users [16]. A higher prevalence of 63% was reported in a study from Barcelona [17]; however, differences in chemsex prevalence may be associated with varying definitions of the term. The Barcelona study included the use of nitrites and erectile dysfunction drugs in its definition of chemsex; typically, only recreational drugs are considered for defining chemsex [15, 16].

Chemsex has consistently been associated with increased CAI incidence [10, 14] and diagnoses of bacterial STIs [10, 16]. The impact of chemsex on STI risk emerged as a critical finding in our research. Participants engaging in chemsex not only exhibited higher rates of STIs but also were more likely to experience reinfections. Moreover, urethral and anal gonococcal infections were found significantly more often in individuals engaging in chemsex practices, potentially facilitated by prolonged and more traumatic sexual activities during chemsex. Additionally, MSM involved in chemsex have been reported to have a greater number of sexual partners and to be more likely to engage in group sex compared to those who do not engage in chemsex practices [18].

Importantly, we found that only a minority, 18% of individuals with 1 or more reinfections, accounted for 68% of all detected STIs. Similar findings were reported by Traeger et al. [4], where 25% of participants accounted for 76% of diagnosed STIs. This indicates that a small group of individuals experienced the majority of STIs, suggesting an opportunity for targeted prevention strategies. We hypothesize that these individuals at highest STI exposure could benefit from more frequent screening. They may also benefit from Doxy PEP, a postexposure prophylaxis containing doxycycline taken within 24–72 h after CAI. The use of Doxy PEP has been shown to significantly decrease the incidence of chlamydia and syphilis infections [19].

In our analysis, most STIs were asymptomatic, yet antimicrobial treatment was initiated following each positive result, in line with current STI treatment guidelines [20]. A low number of symptomatic infections at extragenital sites is commonly observed. In our study, only one in four individuals with anal gonorrhea or chlamydia reported symptoms, which aligns with previously reported numbers ranging from 15–25% [21, 22]. Interestingly, we observed an asymptomatic course of disease in 44% of urethral gonorrhea. Conventionally, symptoms of urethral *N. gonorrhoeae *infections in men are expected in more than 90% of cases [23]. Notably, previous studies predominantly focused on testing individuals with symptomatic urethritis and not on screening. It can be expected that an upscaling of screening for infections will also increase the proportion of asymptomatic infections detected. In this context, the frequency of screening for *N. gonorrhoeae* and *C. trachomatis* as well as the necessity for treatment and associated harm have recently sparked debate. Williams et al. discussed the potential benefits of reducing the screening frequency for gonococcal and chlamydial infections to every 6 or 12 months, while maintaining quarterly screenings for HIV and syphilis, given their higher risk of complications when undiagnosed. Their analysis suggested potential advantages of this approach, including reduced antibiotic exposure, fewer individual adverse effects (such as microbiome disruption or *Clostridium difficile* infection), decreased antimicrobial resistance due to lower antibiotic use at a population level, and cost and resource savings for healthcare systems [24]; however, reducing the screening frequency might have potential drawbacks, including unknown risks of long-term asymptomatic infections by gonorrhea and chlamydia and the potential to transmit these pathogens to women, in whom these infections may potentially cause complications like pelvic inflammatory disease or infertility. The authors concluded that a reduction in screening frequency for chlamydia and gonorrhea would be favorable overall, a conclusion also supported by Kenyon et al. in a recent comprehensive review [25].

While modeling studies have suggested that frequent STI screening might reduce incidence rates [26–28], empirical studies on chlamydia and gonorrhea prevalence have failed to meet these predictions [29, 30]. For instance, the Gonoscreen study, a randomized, controlled trial enrolling two groups of approximately 500 MSM using PrEP, compared the effects of quarterly screening vs. no screening on STI incidence. The authors described a significant reduction in chlamydia infections with screening, while gonorrhea rates remained unchanged; however, the authors discussed that the increased chlamydia incidence in the no screening group was attributed to persistent infections rather than novel infections. In a secondary analysis, correcting for chlamydia positivity at consecutive visits, incidence rates in both groups were comparable [30]. During our observational period, the STI incidence remained stable although individuals were frequently screened. Given the relatively short follow-up period of our study, with 263 participants representing 379 person-years of observation, we cannot draw any conclusions regarding the impact of STI screening on incidence rates.

As strengths of our study, we want to highlight the prospective observational setting and consecutive follow-up, the quarterly STI screening and the detailed information on chemsex use. Importantly, our research provides the very first insights into STI incidence among PrEP users in Austria; however, the study has limitations, including its single-center design, potential bias in self-reported data and a possible selection bias, as it was conducted at a tertiary care hospital.

In conclusion, our study reports on the high incidence of bacterial STIs among PrEP users in Austria, particularly among those involved in chemsex. The absence of new HIV infections demonstrates the effectiveness of PrEP. The recent decision by Austrian healthcare providers to reimburse PrEP [12] marks a significant step towards improving access to HIV prevention. Continued efforts should focus on including regular HIV and syphilis screening in PrEP users. The high number of asymptomatic infections in our study suggests a need to rethink current diagnostic and therapeutic strategies. These measures not only support the overall well-being of PrEP users but also help reduce HIV transmission rates and the overuse of antimicrobial treatment, potentially mitigating the development of antimicrobial resistance.

## Supplementary Information

Details on risk behavior and chemsex use during the study period
