# Supplementary material for: Recurrence of sexually transmitted infections is commonly found in a subpopulation of Austrian users of HIV pre-exposure prophylaxis
Source: Wien Klin Wochenschr. 2025 Mar 2;137(17-18):571–8. doi: 10.1007/s00508-025-02499-6 (PMC12446104; doi:10.1007/s00508-025-02499-6)
Supplement: Supplementary file 1 — Details on risk behavior and chemsex use during the study period [file 508_2025_2499_MOESM1_ESM.docx]

**Table-S1.** Questionnaire addressing risk behavior of the last 12 months at baseline

|  | All Individuals  N=344* | No STIs  N=197 | Diagnosed with at least one STI  N=147 | p-value |
| --- | --- | --- | --- | --- |
| Median number of estimated sex partners (IQR) | 10 (15) | 8 (14) | 14 (19) | **<0.001** |
| Sex practices (%, n/all) |  |  |  |  |
| Oral Sex |  |  |  | 1 |
| Passive | 3% (10/342) | 4% (7/197) | 2% (3/145) | 0.695 |
| Active | 6% (21/342) | 7% (13/197) | 6% (8/145) |  |
| Active & Passive | 91% (311/342) | 90% (177/197) | 92% (134/145) |  |
| Condomless anal intercourse |  |  |  |  |
| Passive | 11% (38/335) | 14% (27/191) | 8% (11/144) | 0.112 |
| Active | 18% (61/335) | 19% (37/191) | 17% (24/144) |  |
| Active & Passive | 70% (236/335) | 66% (127/191) | 76% (109/144) |  |
| Fisting |  |  |  |  |
| Passive | 16% (11/69) | 18% (6/34) | 14% (5/35) | 0.835 |
| Active | 57% (39/69) | 53% (18/34) | 60% (21/35) |  |
| Active & Passive | 28% (19/69) | 29% (10/34) | 26% (9/35) |  |
| Anal bleeding after intercourse (%, n/all) | 44% (149/339) | 40% (76/192) | 50% (73/147) | 0.064 |
| Substance use (%, n/all) |  |  |  |  |
| ‘Poppers’ | 65% (224/344) | 63% (124/197) | 68% (100/147) | 0.328 |
| PDE-5 inhibitors | 36% (122/343) | 30% (59/196) | 43% (63/147) | 0.015 |
| Chemsex | 39% (134/344) | 33% (65/197) | 47% (69/147) | **0.009** |
| Nasal use | 87% (113/130) | 83% (53/64) | 91% (60/66) | 0.171 |
| Oral use | 69% (85/124) | 65% (40/62) | 73% (45/62) | 0.334 |
| Intravenous use | 2% (2/110) | 0% (0/55) | 4% (2/55) | 0.495 |
| Cocaine | 57% (76/134) | 60% (39/65) | 54% (37/69) | 0.457 |
| MDMA | 32% (43/134) | 28% (18/65) | 36% (25/69) | 0.290 |
| Ketamine | 21% (28/134) | 18% (12/65) | 23% (16/69) | 0.501 |
| GHB/GBL | 30% (40/134) | 22% (14/65) | 38% (26/69) | **0.041** |
| Crystal-Meth | 13% (17/134) | 17% (11/65) | 9% (6/69) | 0.153 |
| Mephedrone | 40% (53/134) | 34% (22/65) | 45% (31/69) | 0.190 |

*Sixteen participants did not return the questionnaire.

Abbreviations: GBL, gamma-Butyrolactone; GHB, gamma-Hydroxybutyric acid; MDMA, 3,4-Methylenedioxymethamphetamine; PDE-5, phosphodiesterase type 5; STI, sexually transmitted infection.

**Table-S2.** The table displays the relative number of sexualized drug use (chemsex) at each documented time point

| Substance use | Base-line | 12W | 24W | 36W | 48W | 60W | 72W | 84W | 96W | 108W | 120W | 132W | 144W | 156W |
| --- | --- | --- | --- | --- | --- | --- | --- | --- | --- | --- | --- | --- | --- | --- |
| **Absolute number of users** | 133 | 65 | 49 | 33 | 31 | 28 | 24 | 21 | 20 | 12 | 13 | 6 | 4 | 3 |
| **Cocaine** | 57 | 49 | 33 | 27 | 42 | 29 | 38 | 33 | 30 | 33 | 31 | 17 | 25 | 0 |
| **MDMA** | 32 | 32 | 29 | 36 | 29 | 18 | 29 | 33 | 15 | 25 | 38 | 17 | 0 | 33 |
| **Ketamine** | 21 | 22 | 27 | 36 | 26 | 14 | 21 | 19 | 10 | 8 | 23 | 0 | 0 | 0 |
| **GHB** | 29 | 32 | 27 | 33 | 26 | 21 | 25 | 24 | 20 | 17 | 23 | 0 | 0 | 0 |
| **Crystal Meth** | 13 | 14 | 12 | 6 | 3 | 4 | 4 | 0 | 5 | 17 | 8 | 0 | 0 | 33 |
| **Mephedrone** | 40 | 55 | 51 | 61 | 48 | 39 | 58 | 52 | 80 | 67 | 77 | 67 | 75 | 33 |

Abbreviations: GHB, Gamma-hydroxybutyrate; MDMA, 3,4-Methylenedioxymethamphetamine; W, week.
